# Integrated proteomics and network analysis identifies protein hubs and network alterations in Alzheimer’s disease

**DOI:** 10.1186/s40478-018-0524-2

**Published:** 2018-03-01

**Authors:** Qi Zhang, Cheng Ma, Marla Gearing, Peng George Wang, Lih-Shen Chin, Lian Li

**Affiliations:** 1 Department of Pharmacology and Center for Neurodegenerative Disease, Emory University School of Medicine, Atlanta, GA 30322 USA; 2 Department of Chemistry Center for Diagnostics and Therapeutics, Georgia State University, Atlanta, GA 30303 USA; 3 Department of Pathology and Laboratory Medicine, Emory University School of Medicine, Atlanta, GA 30322 USA

**Keywords:** Alzheimer’s disease, Brain proteome, Mass spectrometry, Quantitative proteomics, Differential expression analysis, Protein co-expression network analysis, Neurodegeneration, Network biology

## Abstract

Although the genetic causes for several rare, familial forms of Alzheimer’s disease (AD) have been identified, the etiology of the sporadic form of AD remains unclear. Here, we report a systems-level study of disease-associated proteome changes in human frontal cortex of sporadic AD patients using an integrated approach that combines mass spectrometry-based quantitative proteomics, differential expression analysis, and co-expression network analysis. Our analyses of 16 human brain tissues from AD patients and age-matched controls showed organization of the cortical proteome into a network of 24 biologically meaningful modules of co-expressed proteins. Of these, 5 modules are positively correlated to AD phenotypes with hub proteins that are up-regulated in AD, and 6 modules are negatively correlated to AD phenotypes with hub proteins that are down-regulated in AD. Our study generated a molecular blueprint of altered protein networks in AD brain and uncovered the dysregulation of multiple pathways and processes in AD brain, including altered proteostasis, RNA homeostasis, immune response, neuroinflammation, synaptic transmission, vesicular transport, cell signaling, cellular metabolism, lipid homeostasis, mitochondrial dynamics and function, cytoskeleton organization, and myelin-axon interactions. Our findings provide new insights into AD pathogenesis and suggest novel candidates for future diagnostic and therapeutic development.

**Electronic supplementary material:**

The online version of this article (10.1186/s40478-018-0524-2) contains supplementary material, which is available to authorized users.

## Introduction

Alzheimer’s disease (AD) is the most common neurodegenerative disorder and the leading cause of dementia in the elderly [53, 70]. Neuropathologically, AD is characterized by the presence of amyloid plaques and neurofibrillary tangles in the brain. The vast majority (95%) of AD cases are sporadic, and the remaining 5% are familial AD [70]. The causative genetic defects for several familial forms of AD have been identified, however, the etiology of sporadic AD remains unknown. The lack of effective means to prevent or treat AD and the failure of recent clinical trials [23, 36, 74] emphasize the need for better understanding AD pathogenic mechanisms to find novel targets for AD therapeutic intervention.

Human postmortem AD brain tissues provide a unique and valuable resource for discovery research to identify specific molecular abnormalities and disease processes associated with sporadic AD. We and others have previously used two-dimensional gel electrophoresis (2-DE)-based proteomics to study differential protein expression in AD versus control brains [17–19, 41]. Although these studies have found some proteins with altered expression in AD [17–19, 41], a limitation of 2-DE proteomics is its relatively low resolution, which limits the number of proteins that can be identified using this approach [6, 30, 79]. Recent advances in high-resolution, high-mass-accuracy mass spectrometry-based proteomics technologies provide powerful, new tools for in-depth profiling and quantitative analysis of protein expression in complex biological samples such as human brain tissues [21, 69].

With the advanced proteomics technologies enabling simultaneous, quantitative measurement of expression profiles for thousands of proteins, how to analyze such large proteomic data sets at the systems level becomes a major challenge. Weighted gene co-expression network analysis (WGCNA) is a systems biology approach originally developed for analysis of high-throughput transcriptomic data to provide an unbiased systems-level organization of the transcriptome into a network of biologically meaningful modules of co-expressed genes [45, 62, 92]. The use of WGCNA in studying transcriptome changes in a number of human diseases has led to the identification of disease-associated network modules and hub genes, which are the most highly connected genes that are key determinants of module function and represent important molecular targets for understanding and treating diseases [12, 27, 33, 46, 54, 82, 88]. Recent studies have begun to show that WGCNA can also be used in analyzing large proteomic data sets to gain systems-level insights into disease-associated proteome changes [37, 71, 80, 93].

In the present study, we performed large-scale, unbiased proteomic analyses of human AD and control frontal cortex tissues to determine disease-associated brain proteome changes by using a liquid chromatography-tandem mass spectrometry (LC-MS/MS)-based, label-free quantitative proteomic approach. In addition to differential expression analysis to identify brain proteins with significantly altered abundance in AD, we performed WGCNA-based systems-level analysis of our entire proteomic data set and identified a network of disease-associated protein modules and intra-modular hub proteins in AD brain. Our study reveals dysregulation of multiple pathways and processes in AD brain and provides novel insights into the pathogenic mechanisms of sporadic AD.

## Materials and methods

### Human brain tissues

Postmortem frontal cortex tissues from neuropathologically confirmed AD cases and age-matched control subjects were obtained from Emory Center for Neurodegenerative Disease Brain Bank. Amyloid plaque pathology was assessed using the Consortium to Establish a Registry for Alzheimer’s Disease (CERAD) protocol for neuritic plaque scoring [57], and neurofibrillary tangle pathology was assessed using the Braak staging system [11]. All AD cases meet the criteria of high level of AD neuropathological change based on the ABC scores according to the National Institute on Aging-Alzheimer’s Association guidelines for the neuropathological assessment of Alzheimer’s disease [58]. ApoE genotypes were determined as previously described [29]. Control subjects had no known history of neurological disease and showed no significant neurodegenerative changes at autopsy. Clinical and neuropathological data of all cases, including age, gender, disease status, age at onset, amyloid plaque pathology, neurofibrillary tangle pathology, ApoE genotype, and postmortem interval, are provided in Additional file 1: Table S1. Power analysis showed that the sample size used in this study (the total number of subjects = 16; *n* = 8 in each AD or control group) has > 80% power at a two-sided Type I error rate of 5% to detect effect size of > 1.6.

### Brain tissue homogenization and protein extraction

Approximately 25 mg of human frontal cortex tissue from each AD or control case was homogenized as described [87] in 150 μl of lysis buffer containing 4% SDS, 100 mM DTT, and 100 mM Tris–HCl, pH 7.6, followed by incubation at 95 °C for 5 min. After cooling to room temperature, the homogenate was centrifuged at 16,000 x g for 5 min to obtain supernatant containing extracted proteins. Because the presence of SDS efficiently inactivates protease activity [87], no protease inhibitors were included during the brain tissue homogenization and protein extraction process. Protein concentrations of brain protein extracts were measured by UV spectrometry at 280 nm with NanoDrop spectrophotometer (ThermoFisher) using an extinction coefficient of 1.1 for 0.1% (g/L) solution [87].

### Filter-aided sample preparation (FASP)

Human brain protein extracts were processed by using the FASP protocol as described [87]. Briefly, 30 μl of each protein extract was mixed with 200 μl of 8 M urea in 100 mM Tris-HCl, pH 8.5 (UA solution), and the mixture was transferred into a Microcon 30-kDa centrifugal filter unit (MRCF0R030, Merck) and centrifuged at 14,000 x g for 15 min. Cysteine residues were alkylated by adding 100 μl of UA solution containing 50 mM iodoacetamide to the filter unit and incubation in darkness for 30 min at room temperature. After centrifugation at 14,000 x g for 10 min, 100 μl of UA solution was added to the filter unit and centrifuged again. This UA washing step was repeated twice, and the filter unit was then washed with 100 μl of 50 mM NH_4_HCO_3_ two times. Next, protein digestion was carried out by adding 40 μl of 50 mM NH_4_HCO_3_ solution containing sequencing-grade trypsin (enzyme to protein ratio 1:100) in the filter unit and incubation at 37 °C for 12 h. Digested peptides were eluted by adding 100 μl of 50 mM NH_4_HCO_3_ and collected by centrifugation at 14,000 x g for 10 min as a filtrate, and this step was repeated five times. The collected peptides were further purified by using a self-packed C18 ZipTip micro-column. The final concentration of peptides was determined by UV-spectrometry using an extinction coefficient of 1.1 for 0.1% (g/L) solution at 280 nm [87]. All peptides were dried under vacuum at room temperature.

### Liquid chromatography-tandem mass spectrometry

LC-MS/MS proteomic analyses were performed using the LTQ-Orbitrap Elite mass spectrometer (ThermoFisher) equipped with an EASY-Spray source and a nano-LC UltiMate 3000 high-performance liquid chromatography system (ThermoFisher). Human brain-derived peptides (2 μg) from each sample were separated by online reversed phase (RP)-HPLC fractionation on an EASY-Spray PepMap C18 column (length, 50 cm; particle size, 2 μm; pore size, 100 Å; ThermoFisher), using a 240-min gradient from 2% to 50% solvent B at a flow rate of 300 nL/min (mobile phase A, 1.95% acetonitrile, 97.95% H_2_O, 0.1% formic acid; mobile phase B, 79.95% acetonitrile, 19.95% H_2_O, 0.1% formic acid). A full-scan survey MS experiment (m/z range from 375 to 1600; automatic gain control target, 1,000,000 ions; resolution at 400 m/z, 60,000; maximum ion accumulation time, 50 ms) was performed using the Orbitrap mass analyzer. The ten most intense ions were selected and fragmented in the LTQ mass spectrometer (automatic gain control target value, 10,000) via collision-induced dissociation (CID) with maximum ion accumulation time of 100 ms. Raw data were analyzed by using Proteome Discoverer 1.4 (ThermoFisher) to search against the human Uniprot TrEMBL database (2016_02 Release, 20,198 reviewed entries). The modifications were set as follows: static modification of carbamidomethyl (Cys, + 57.0214 Da); dynamic modification of deamination (Asn, + 0.9840 Da), oxidation (Met, + 15.9949 Da), and acetylation (Lys, + 42.0106 Da). Trypsin was selected as the proteolytic enzyme, and up to two missed cleavages were allowed. The mass tolerance was set to 20 ppm for the precursor ions and 0.5 Da for the fragment ions. The false discovery rate (FDR) for peptide and protein identification was set to 1%.

### Label-free protein quantification

Label-free protein quantitative analysis was performed by using Proteome Discoverer 1.4 to quantify precursor ion peak area (i.e., area under the curve), which is linearly proportional to protein abundance [21]. A limitation of “shotgun” label-free quantitative proteomics is that protein identification or abundance data can be missing in some samples [35]. Therefore, we restricted quantitative analysis to the proteins with complete data in all 16 brain samples, excluding proteins with missing data in any sample. In each sample, relative protein abundance for each protein was determined by normalizing the peak area of the protein to the total peak area of all proteins in the sample as described [86]. To account for technical variability present in filter-aided sample preparation and LC-MS/MS analyses, each protein extract was spiked with bovine alpha-2-HS-glycoprotein (fetuin) at 0.1% (μg/μg total protein) as an internal control. The relative protein abundances determined by normalizing each protein peak area to that of the spike-in fetuin protein were similar to the abundances determined by normalizing to the total protein peak area, confirming the validity of the ‘Total Protein Approach’-based protein quantification analysis [86]. The technical variation of the FASP sample processing and LC-MS/MS quantification system, estimated from the relative abundances of the spike-in fetuin protein after normalization of its peak area to the total protein peak area in eight control brain samples, had a coefficient of variation (CV) of 6%.

### Differential expression analysis

Differentially expressed proteins in AD versus control were identified by using unpaired two-tailed Student’s *t* test with the thresholds of ±1.3-fold change over the control (i.e., AD/control ratio > 1.3 or < 0.77) and a *P* value < 0.05. The *q* values were calculated by using the q value R package [76] to correct for multiple comparisons and estimate the false discovery rates [77]. Significantly altered proteins in AD with corresponding *P* values and *q* values are provided in Additional file 2: Table S2.

### Hierarchical clustering analysis

Unsupervised hierarchical clustering of individual clinical cases and the identified differentially expressed proteins was performed based on their relative protein abundances in each samples by using Heatmapper online tool with an average linkage clustering and Kendall’s tau distance measurement method [4]. Protein expression heat map with dendrograms showing clustering results were generated and visualized by the Heatmapper.

### Protein co-expression network analysis

Protein co-expression network analysis was performed with the R package WGCNA as described [45] using the entire proteomic data set of all identified proteins with no missing values. Briefly, a correlation matrix for all pair-wise correlations of proteins across all samples was generated and then transformed into a matrix of connection strengths, i.e., a weighted adjacency matrix, as described [45, 92] with a soft threshold power β = 16. The connection strengths were then used to calculate topological overlap (TO), a robust, pairwise measure which indicates two proteins’ similarity based on their co-expression relationships with all other proteins in the network [90]. Proteins were hierarchically clustered using 1 − TO as the distance measure to generate a cluster dendrogram, and modules of proteins with similar co-expression relationships were identified by using a dynamic tree-cutting algorithm [47] with the following parameters: minimal module size = 23, deepSplit = 4, and merge cut height = 0.07. For each module, a module eigenprotein was defined as the first principal component of the module which is a weighted summary of protein expression in the module and explains the maximal possible variability for all proteins within the module [32]. Module membership (kME) was determined by calculating Pearson correlation between each protein and each module eigenprotein and the corresponding *P*-values [32]. Proteins were (re)assigned to the module for which they had the highest module membership with a reassignment threshold of *P* < 0.05. Module-trait relationships were determined by using the WGCNA package [45] to calculate the biweight midcorrelations between each module eigenprotein and a clinical or neuropathological trait and the corresponding *P*-values. Module networks were graphically depicted by using the igraph package in R [61].

### Gene ontology enrichment analysis and functional annotation of modules and proteins

Gene ontology (GO) enrichment analysis of the generated datasets of differentially expressed proteins and WGCNA module proteins was performed using MetaCore bioinformatics software (Version 6.29, build 68,613; https://portal.genego.com/). The total list of all proteins identified in human frontal cortex samples was used as the background. The hypergeometric test after the Benjamini-Hochberg false discovery rate (FDR) correction was used to assess statistical significance. Enriched GO terms with FDR-corrected *P* < 0.05 were considered statistically significant. In addition to the use of functional annotation tools, we also searched PubMed manually to gain insights into the functions of the identified differentially expressed proteins and WGCNA module proteins.

### Western blot analysis

Human frontal cortex tissues from individual AD or control cases were homogenized in SDS lysis buffer, and protein extracts were subjected to SDS-PAGE. The proteins were then transferred onto PVDF membranes (EMD Millipore) and probed with anti-Smac antibody (1:1000 dilution; Cell Signaling Technology), anti-STK39 antibody (1:1000 dilution; Cell Signaling Technology), or anti-β-actin antibody (1:5000 dilution; EMD Millipore) followed by horseradish-peroxidase-conjugated secondary antibodies (Jackson ImmunoResearch Laboratories) and visualization using enhanced chemiluminescence as described previously [48]. The expression levels of each protein were quantified by measuring protein intensities on immunoblot images using the Image J software (National Institutes of Health) and normalized to the corresponding level of β-actin in each sample. The normalized protein abundances across AD and control cases were compared by using unpaired two-tailed Student’s *t* test, and *P* < 0.05 was considered statistically significant.

## Results

### Analysis of AD-associated proteome changes by quantitative proteomics

To investigate brain proteome alterations associated with sporadic AD, we analyzed brain samples from eight clinically and neuropathologically characterized AD patients and eight age-matched control subjects (Additional file 1: Table S1). Proteins were extracted from the dorsolateral prefrontal cortex tissues of these individuals by using the detergent sodium dodecyl sulfate (SDS) because it is the most effective reagent for solubilizing tissues and cells to achieve complete extraction of proteins [87]. We used a recently developed, filter-aided sample preparation (FASP) method [85, 87] for detergent removal and protein digestion to obtain high-purity peptides from the brain samples. Subsequent LC-MS/MS proteomic analysis using the high-resolution high-mass-accuracy LTQ-Orbitrap Elite mass spectrometer identified a total of 39,819 distinct peptides, corresponding to 6679 unique proteins. Due to stochastic nature of “shotgun” label-free quantitative proteomics, protein identification or abundance data are sometimes missing in certain samples [35]. The proteins with missing data in any sample were excluded in our analysis, resulting in the final quantification of 1968 proteins with complete data across all 16 brain samples from AD and control cases (Additional file 2: Table S2).

### Differential expression analysis identifies proteins with altered abundance in AD

**Fig. 1:**
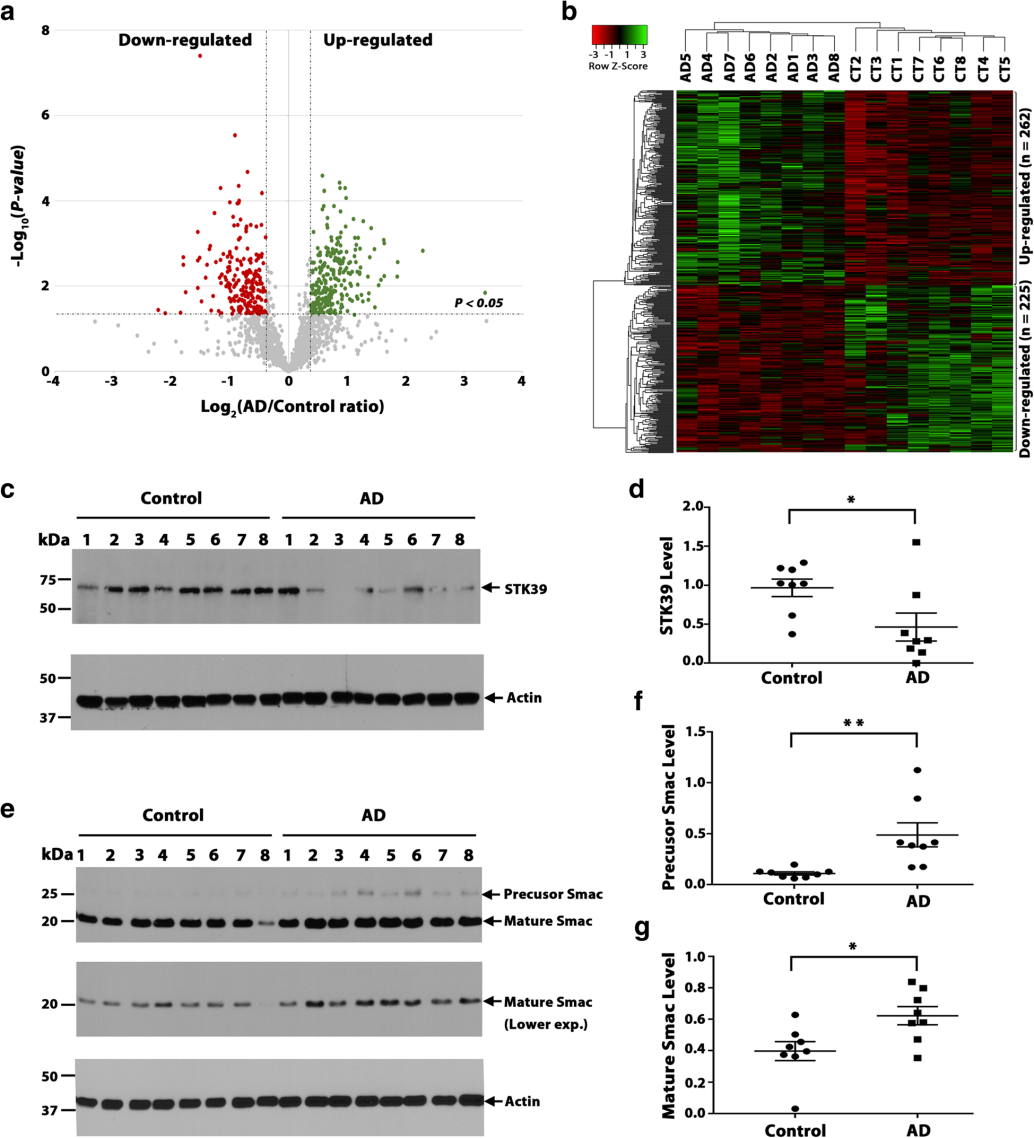
AD-associated brain proteome changes revealed by label-free quantitative proteomics. **a** Volcano plot displaying the distribution of all proteins (*n* = 1968) with relative protein abundance (log_2_ AD/control ratio) plotted against its significance level (negative log_10_ *P*-value), showing significantly (*P* < 0.05) increased (AD/control ratio > 1.3; Green) and decreased (AD/control ratio < 0.77; Red) proteins in AD. **b** Heat map representation of 16 individual sample abundances for 487 significantly altered proteins after unsupervised hierarchical clustering, segregating samples into AD (left) and controls (CT; right) and proteins into up-regulated (top) and down-regulated (bottom) proteins in AD. **c-g** Western blot analysis (c, e) and quantification (d, f, g) confirm the decreased expression of STK39 (c, d) and increased expression of Smac proteins (e-f) in AD versus control. Data represent mean ± SEM (error bars; *n* = 8 biological repeats for AD or control group). *, *P* < 0.05; **, *P* < 0.01, unpaired two-tailed Student’s *t* test. Each experiment was repeated three times with similar results

We performed differential expression analysis of quantitative proteomics data using the thresholds of ±1.3-fold change in AD over the control (*P* < 0.05) and identified 487 differentially expressed proteins (262 up-regulated proteins and 225 down-regulated proteins) in AD at FDR < 0.11 (Fig. 1a and Additional file 2: Table S2). Unsupervised hierarchical clustering analysis based on the protein abundances in the 16 individual brain samples showed that the identified differentially expressed proteins can serve as a proteomic signature for distinguishing AD versus control cases (Fig. 1b). The heat map illustrated an overall reproducibility as well as individual heterogeneity of protein expression profiles among different subjects within the AD or control group (Fig. 1b).

The list of the identified dysregulated proteins in AD (Additional file 2: Table S2) includes a number of proteins that have been previously shown by our group and others to be differentially expressed in AD brain, such as DJ-1, APOE, clusterin (CLU), and UCH-L1 [1, 17, 19, 55]. In addition, our proteomic analysis also identified 322 novel proteins that have not been previously reported as differentially expressed in AD, such as serine/threonine protein kinase 39 (STK39) and DIABLO/Smac (Additional file 3: Table S3). To validate our proteomic results, we performed Western blot analysis of STK39 and Smac expression in AD and control brains (Fig. 1c-g). We found that, in accordance with the proteomic data (Additional file 2: Table S2), STK39 protein level was significantly decreased in AD versus control (Fig. 1c, d). STK39 is an important kinase that has been associated with hypertension, Parkinson’s disease, and autism [50, 67, 84]. Our results indicate, for the first time, a link between STK39 and AD. In addition, our Western blot analysis also validated Smac, a key regulator of apoptosis [40], as an up-regulated protein in AD brain (Fig. 1e-f). Together, these results provide support for the robustness of our label-free quantitative proteomic analysis.

**Fig. 2:**
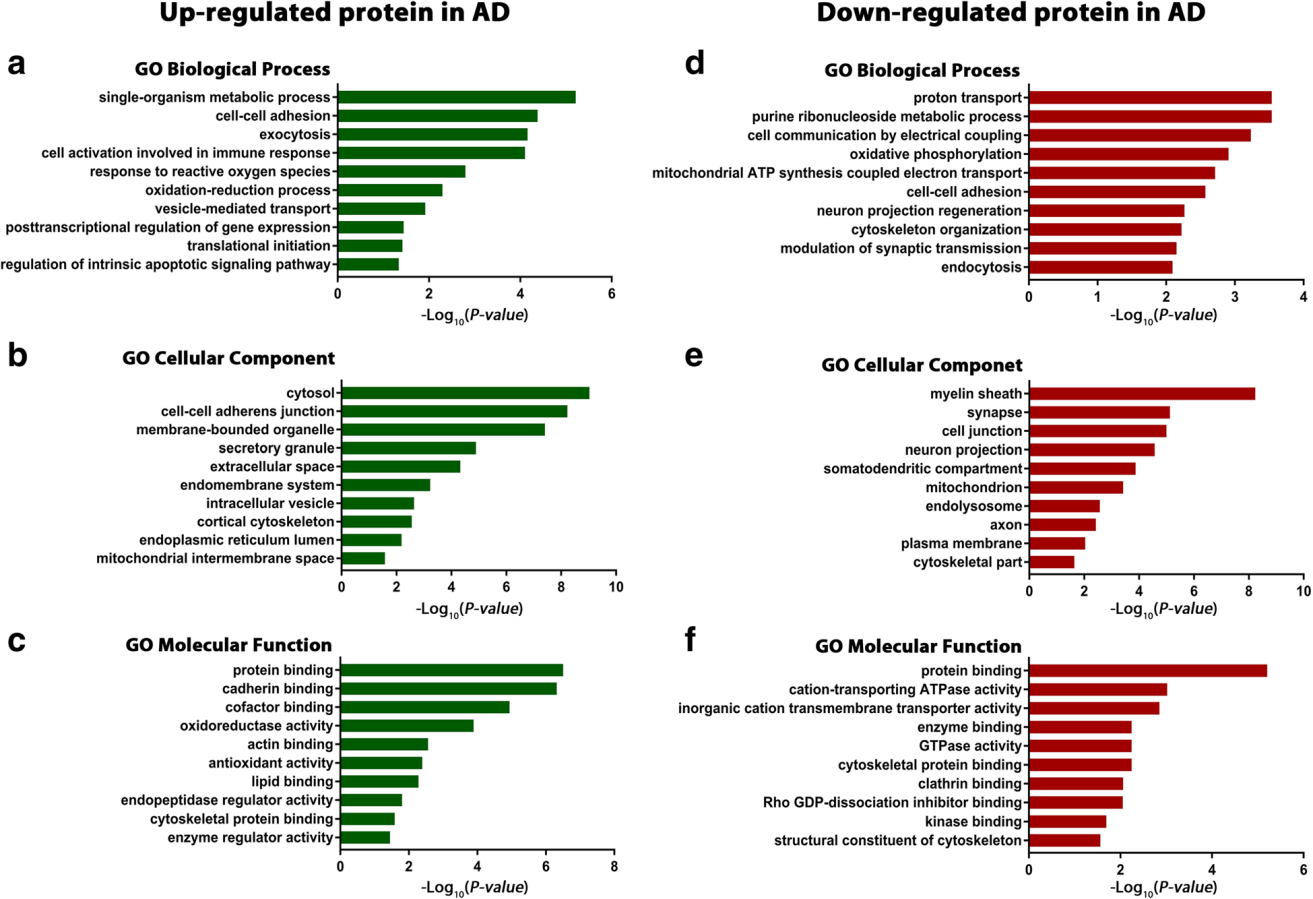
Gene ontology enrichment analysis of differentially expressed proteins in AD brain. GO biological process, cellular component, and molecular function enrichment analyses of up-regulated (**a**-**c**) and down-regulated (**d**-**f**) proteins in AD were performed using MetaCore bioinformatics software. Significantly enriched GO terms are shown with Benjamini-Hochberg FDR-corrected *P*-values

Next, we performed gene ontology (GO) enrichment analysis of the identified differentially expressed proteins to gain insights into the cellular functions and biological processes that are affected in AD brain (Fig. 2; Additional file 4: Table S4). We found that down-regulated proteins in AD were significantly enriched with GO categories linking to ion transport, mitochondrial function, synaptic transmission, myelin sheath, cell-cell adhesion, cytoskeleton organization, and endocytosis, whereas up-regulated proteins in AD were overrepresented with GO terms associated with metabolic process, immune response, cell-cell adhesion, exocytosis, vesicle-mediated transport, response to oxidative stress, translation, and regulation of apoptotic signaling (Fig. 2; Additional file 4: Table S4).

### Co-expression network analysis uncovers AD-associated protein network alterations

**Fig. 3:**
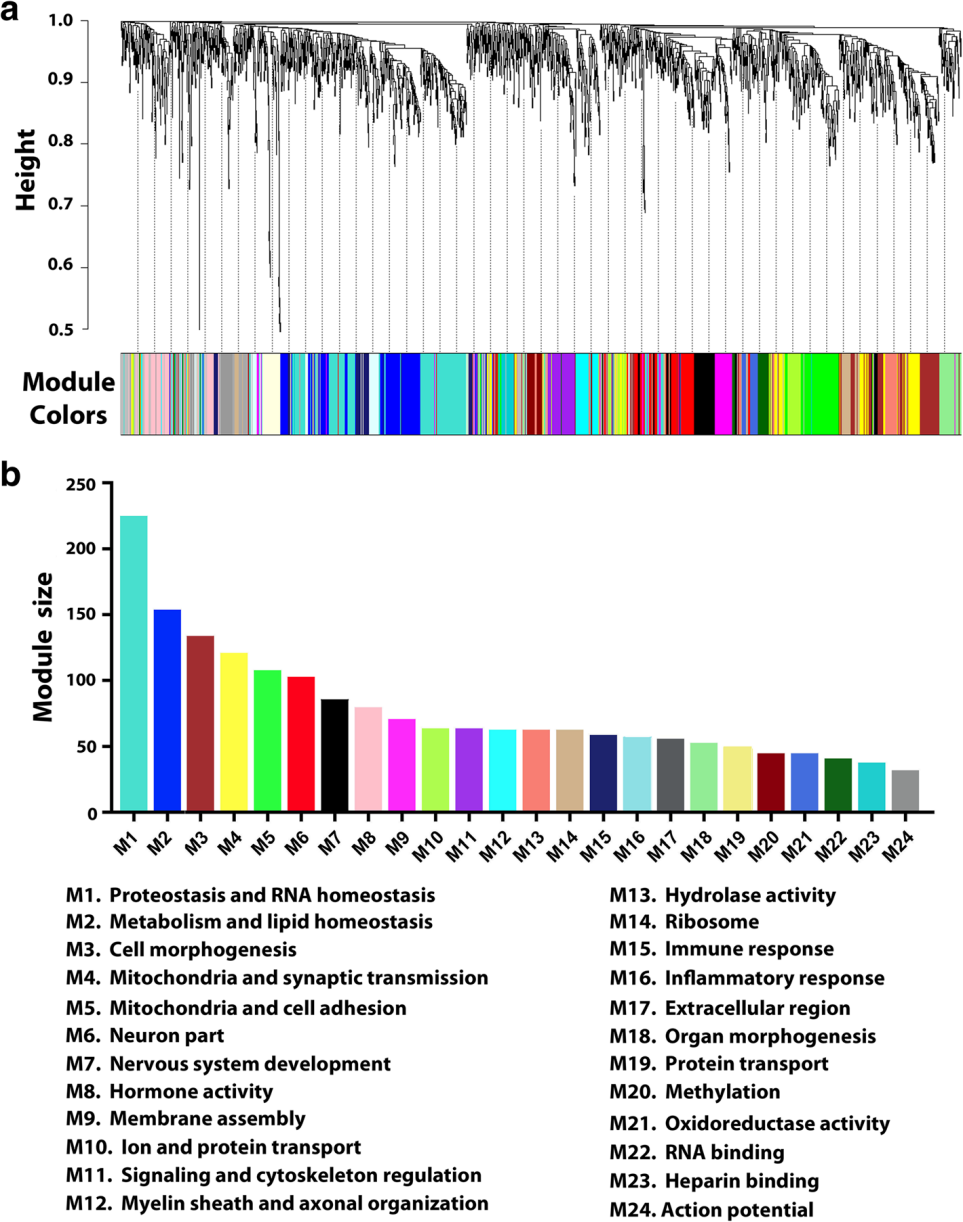
Protein co-expression network analysis organizes the brain proteome into biologically meaningful modules. **a** WGCNA cluster dendrogram generated by unsupervised hierarchical clustering of all proteins in the entire proteomic data set on the basis of topological overlap followed by branch cutting reveals 24 network modules coded by different colors. **b** Protein co-expression modules were assigned M1 to M24 based on their module size. Representative functional categories enriched in these modules are indicated below the graph

To gain systems-level insights into the brain proteome changes in AD, we performed protein co-expression network analysis by using WGCNA, a data-driven network approach which uses pairwise correlation relationships of proteins and their topological overlap to organize the proteome into a network of biologically meaningful modules of co-expressed proteins [45, 90, 92]. We applied WGCNA to our entire proteomic data set of all proteins with no missing values (*n* = 1968 proteins) and constructed a protein co-expression network from protein expression profiles across all AD and control samples. Our WGCNA analysis identified 24 network modules of strongly co-expressed proteins (Fig. 3a; Additional file 5: Table S5). These modules, color coded according to the convention of WGCNA [45, 92], were labeled M1 to M24 based on the module size, ranging from the largest (M1: 223 proteins) to the smallest (M24: 30 proteins) (Fig. 3b). We found that several modules were significantly enriched for brain-specific GO categories, including mitochondria and synaptic transmission (M4), neuron part (M6), nervous system development (M7), myelin sheath and axonal organization (M12), and action potential (M24), whereas other modules were associated with GO categories linked to discrete cellular structures and functions, such as proteostasis and RNA homeostasis (M1), metabolism and lipid homeostasis (M2), cell morphogenesis (M3), mitochondria and cell adhesion (M5), hormone activity (M8), membrane assembly (M9), ion and protein transport (M10), signaling and cytoskeleton regulation (M11), hydrolase activity (M13), ribosome (M14), immune response (M15), inflammatory response (M16), and extracellular region (M17) (Fig. 3b; Additional file 6: Table S6).

**Fig. 4:**
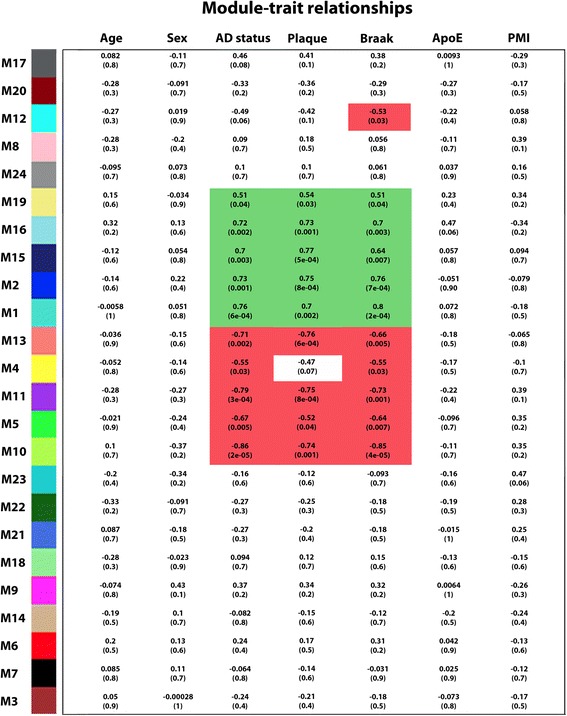
Identification of disease-relevant protein modules associated with AD phenotypic traits. Module-trait relationships were determined by biweight midcorrelation between module eigenprotein expression and the indicated clinical or neuropathological feature. Correlation coefficients are indicated on the top with corresponding *P*-values in brackets below. Significant positive correlations (cor > 0.50, *P* < 0.05) are highlighted in *Green*, and significant negative correlations (cor < − 0.50, *P* < 0.05) are in *Red*. PMI, postmortem interval

**Fig. 5:**
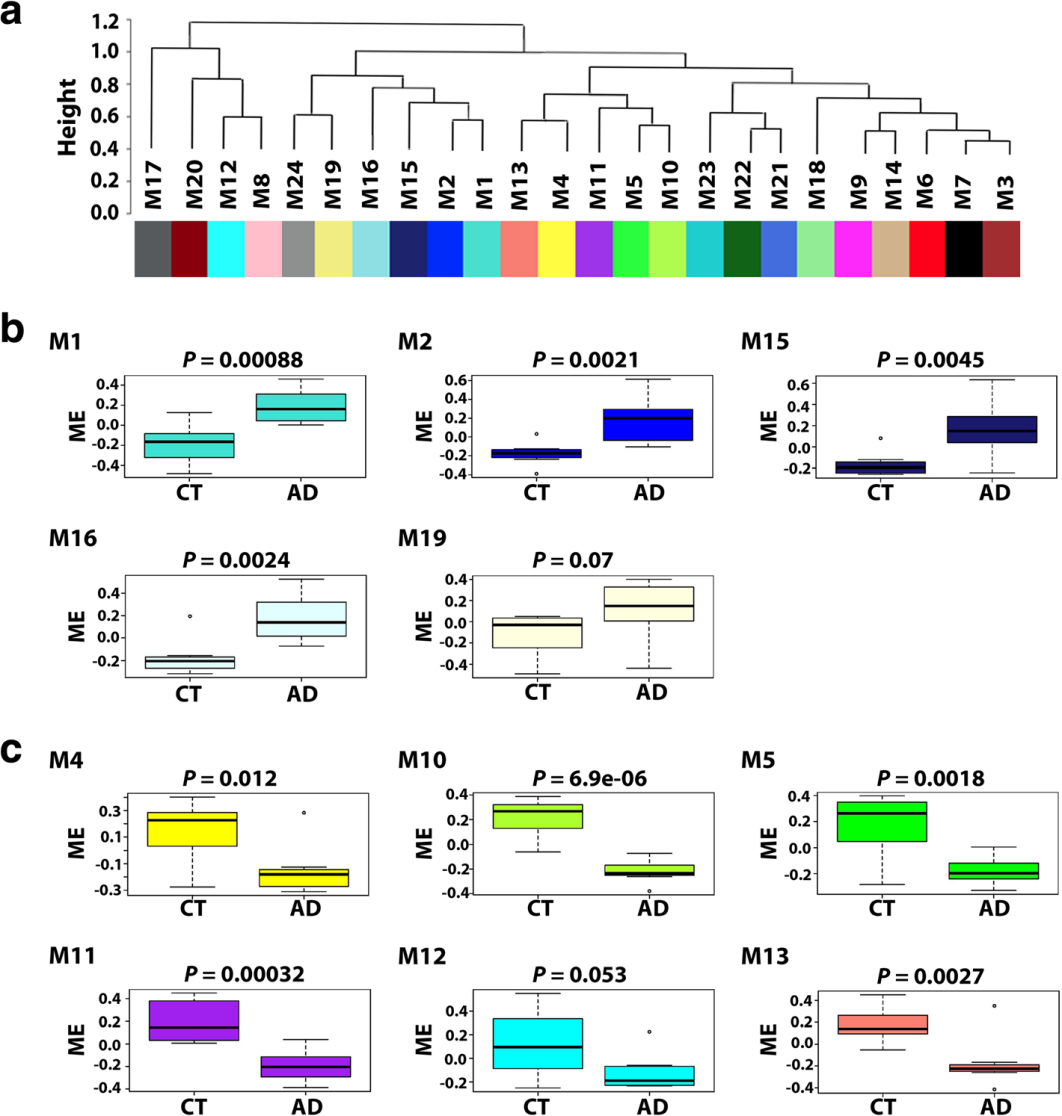
Inter-modular relationships and module expression profiles of AD-related modules. **a** Module eigenprotein meta-network showing the inter-modular relationships of the identified 24 protein co-expression modules. **b** Box plots showing module eigenprotein (ME) values in AD and control (CT) cases for modules that are positively correlated with AD phenotypes. **c** Box plots showing ME values in AD and CT cases for modules that are negatively correlated with AD phenotypes. Box plots depict the mean (horizontal bars) and variance (25th to 75th percentiles), and significance (*P*-value) of differential ME expression in AD versus control was determined using unpaired two-tailed Student’s *t* test

To identify disease-relevant modules associated with AD phenotypic traits, we assessed the module-trait relationships by determining the biweight midcorrelations between each module eigenprotein (the module representative which summarizes protein expression profiles in the module [32]) and various disease-related traits or sample variables (Fig. 4). We identified 11 modules that were significantly correlated with AD status, amyloid plaque pathology (frontal cortex neuritic plaque frequency), and/or neurofibrillary tangle pathology (Braak stage), including 5 positive correlated modules (M1, M2, M15, M16, and M19) and 6 negatively correlated modules (M4, M5, M10, M11, M12, and M13). None of the modules showed significant correlation with age, gender, ApoE genotype, or postmortem interval (Fig. 4), confirming that the identified AD-correlated modules are not due to any of the potential confounding factors. Our analysis showed that most of the positively correlated modules (M1, M2, M15, and M16) had significantly increased module expression levels in AD (Fig. 5b), whereas most of the negatively correlated modules (M4, M5, M10, M11, and M13) had significantly decreased module expression levels in AD (Fig. 5c).

We then assessed the inter-modular relationships by performing eigenprotein network analysis as described [32, 44] to construct a higher-order meta-network based on pairwise correlation relationships of module eigenproteins. The module eigenprotein meta-network revealed the inter-modular connectivity of 24 co-expression modules in brain proteome, showing a hierarchical organization of highly interconnected modules into meta-modules, i.e., groups of highly correlated module eigenproteins (Fig. 5a). Interestingly, the eigenproteins of all modules positively correlated with AD phenotypes (M1, M2, M15, M16, and M19) were clustered in a single meta-module (Fig. 4 and Fig. 5a), suggesting close relationships among the pathways and processes associated with these positively correlated modules. In addition, we identified a meta-module containing eigenproteins from 5 out of the 6 modules negatively correlated with AD phenotypes (M4, M5, M10, M11, and M13), indicating that the corresponding pathways and processes for these negatively correlated modules may also be related.

### AD-associated network modules and hub proteins reveal multiple dysregulated pathways in AD brain

**Fig. 6:**
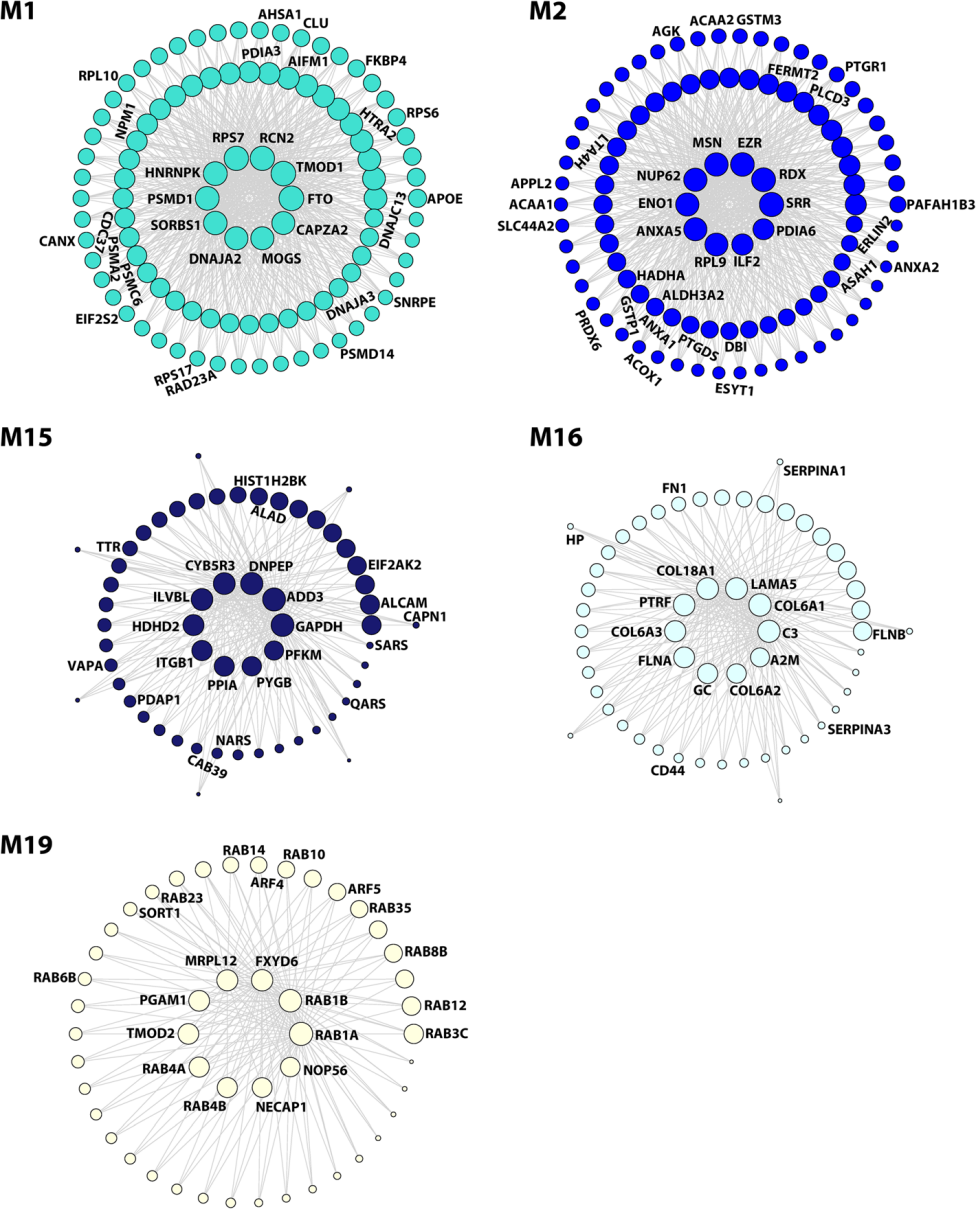
Network depiction of protein co-expression modules that are positively correlated with AD pathology. Nodes represent proteins and edges (lines) indicate connections between the nodes, with a maximum of top 100 proteins and top 700 connections shown for each module. The size of the nodes corresponds to the intramodular connectivity as measured by kME. The top 10 highly connected hub proteins are shown in the center of each network plot. Proteins that are mentioned in the *Results* section are indicated. The complete list of proteins in each module and their kME values are provided in Additional file 5: Table S5

**Fig. 7:**
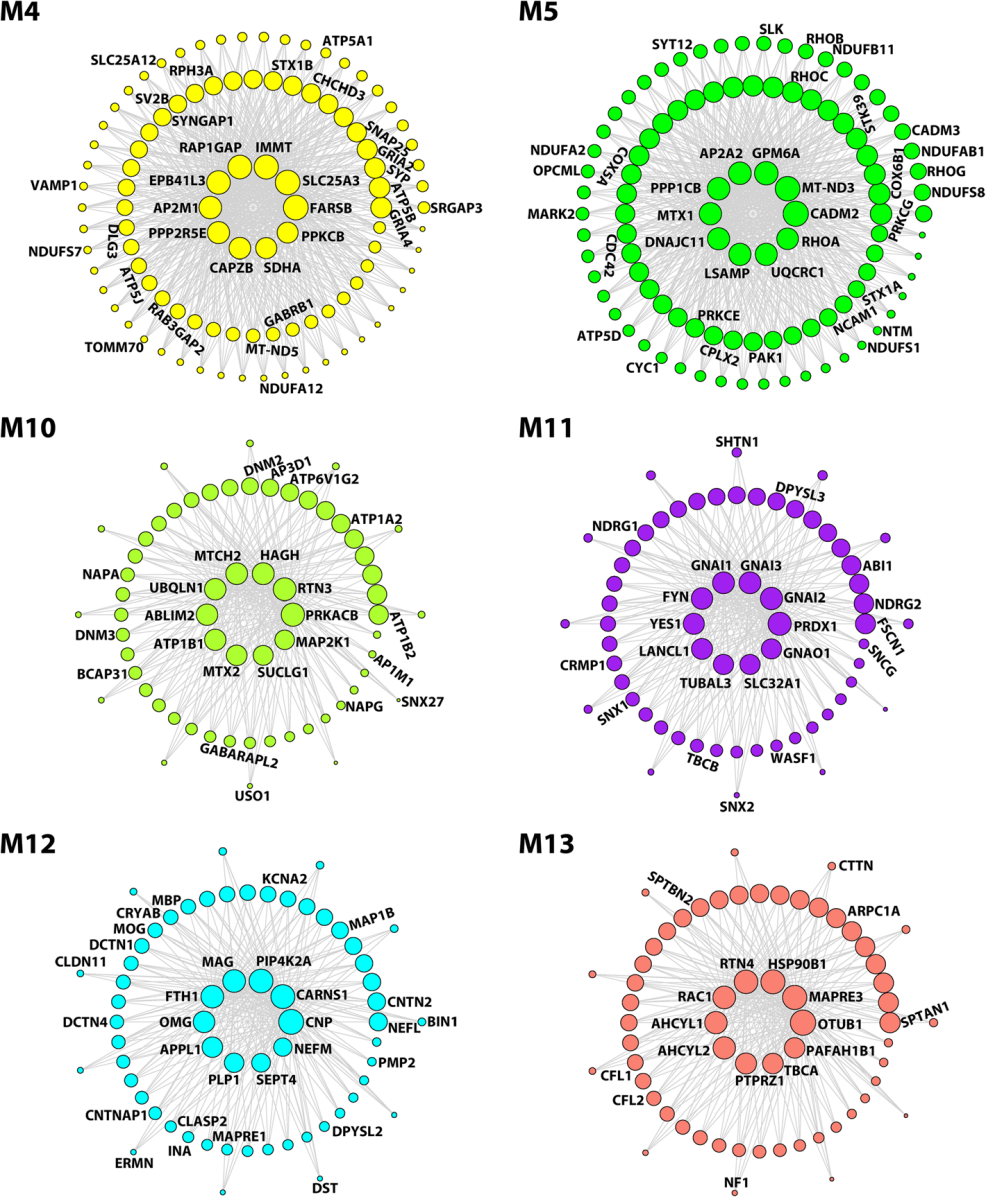
Network depiction of protein co-expression modules that are negatively correlated with AD pathology. Nodes represent proteins and edges represent connections, with a maximum of top 100 proteins and top 700 connections shown for each module. The size of the nodes corresponds to the intramodular connectivity as measured by kME. The top 10 highly connected hub proteins are shown in the middle of each network plot. Proteins that are mentioned in the *Results* section are indicated. The complete list of proteins in each module and their kME values are provided in Additional file 5: Table S5

**Fig. 8:**
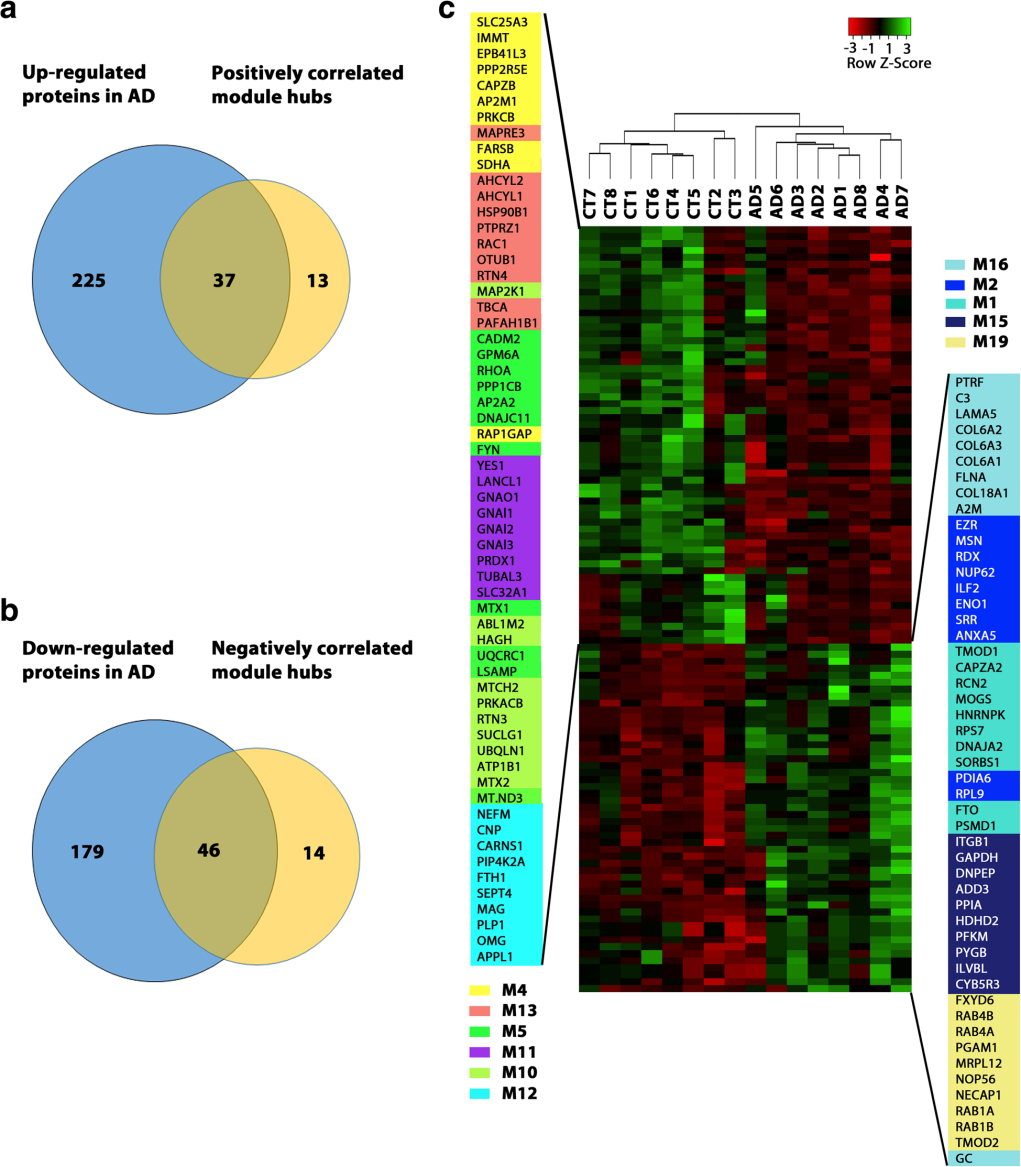
Hub proteins of AD-related modules provide a molecular signature for differentiating AD and control cases. **a** Venn diagram showing the overlap between the identified up-regulated proteins in AD and top intramodular hub proteins of co-expression modules with positive correlation to AD phenotypes. **b** Venn diagram showing the overlap between the identified down-regulated proteins in AD and top intramodular hub proteins of co-expression modules with negative correlation to AD phenotypes. **c** Heat map representing the expression profiles of 110 highly connected intramodular hub proteins after unsupervised hierarchical clustering, showing clear separation of AD from control (CT) cases and positively correlated module hub proteins from negatively correlated module hub proteins

Highly connected hub nodes are central to a network’s architecture and function [2, 7], and intramodular hub proteins in disease-related WGCNA modules have emerged as key targets for biomarker and therapeutic development [12, 27, 33, 46, 54, 82, 88]. Intramodular hub proteins can be identified by using module membership (kME), a measure of intramodular connectivity [32, 46]. The top 10 highly connected hub proteins for each of the identified AD-related modules are shown in the center of network plots (Figs. 6 and 7). Unsupervised hierarchical clustering analysis based on the hub protein expression profiles showed that the identified top hub proteins serve as a molecular signature to differentiate AD and control cases (Fig. 8c). We found that the top hub proteins of the modules with positive correlation to AD phenotypes were often up-regulated in AD (Fig. 8a,c), whereas the top hub proteins of the negative correlated modules were often down-regulated in AD (Fig. 8b,c), consistent with the proposed role of hub proteins as key drivers of protein co-expression modules [32, 33]. We assessed the molecular and functional characteristics of each AD-associated module based on its top hub proteins and gene ontology enrichment analysis of module proteins to gain insights into the biological roles of AD-related modules (Additional file 6: Table S6).

Our analyses revealed that M1, the largest module positively correlated with AD phenotypes (Fig. 4), was significantly enriched with GO categories and hub proteins linked to pathways that control protein homeostasis, or “proteostasis” (Fig. 6 and Additional file 6: Table S6), including 11 protein translation machinery components (EIF2S2, EIF3A, EIF4A2, EIF4B, RPLP1, RPL3, RPL10, RPS6, RPS7, RPS14, and RPS17) with 40S ribosome subunit RPS7 as a top hub protein; 19 molecular chaperones and cochaperones (AHSA1, CDC37, BAG5, CANX, DNAJA2, DNAJA3, DNAJC13, FKBP4, ERO1A, GNB4, GANAB, PDIA3, PFDN5, PFDN6, TBCD, CCT4/TCP1-delta, CCT5/TCP1-epsilon, CCT6A/TCP1-zeta-1, and CLU) with Hsp70 cochaperone DNAJA2 as a top hub protein; and 11 proteasome complex components (PSMA2, PSMC1, PSMC2, PSMC4, PSMC6, PSMD1, PSMD12, PSMD13, PSMD14, RAD23A, and RAD23B) with 26S proteasome regulatory subunit PSMD1 as a top hub protein (Fig. 6 and Additional file 5: Table S5). The overrepresentation of the proteostasis machinery components in this AD-related module supports the involvement of dysregulated proteostasis in AD pathophysiology [43, 78, 89].

In addition to proteostasis, RNA homeostasis-related proteins and pathways were also enriched in the M1 module, as demonstrated by the presence of 12 ribonucleoproteins involved in RNA processing (HNRNPC, HNRNPK, HNRNPL, ALYREF, GCN1L1, SSB, NPM1, LUC7L3, TROVE2, EFTUD2, RUVBL1, and SNRPE) with heterogeneous nuclear ribonucleoprotein K (HNRNPK) as a top hub protein (Fig. 6 and Additional file 5: Table S5). Our finding of HNRNPK, a major RNA-binding protein which functions in regulation of transcription, RNA splicing, mRNA stability, and translation [9], as an up-regulated M1 hub protein in AD (Fig. 8) reveals a previously unrecognized role of HNRNPK in AD pathophysiology. Corroborating with our results, another related M1 module member, HNRNPC, has been reported to be increased in AD and promote APP translation [10, 66]. Additionally, we identified pro-apoptotic factors HTRA2 and AIFM1 as top hub proteins up-regulated in AD (Fig. 6, Additional file 2: Table S2, and Additional file 5: Table S5), indicating enhanced apoptotic signaling is another key feature of this module.

The relevance of the M1 module to AD is further strengthened by its association with APOE and CLU (Fig. 6), two well-established, genetic risk factors for sporadic AD [22]. Our analyses showed that both APOE and CLU proteins were up-regulated in AD (Additional file 2: Table S2) and had high intramodular connectivity values (Additional file 5: Table S5), supporting their role as important determinants of M1 module functions. In addition, we found the fat mass and obesity-associated protein FTO, an AD risk factor which genetically interacts with APOE [38, 68], was the most highly connected hub protein of the M1 module (Fig. 6 and Additional file 5: Table S5). FTO, a demethylase which regulates 6-methyladenosine modifications of mRNAs, has also been linked to increased risk for obesity and type 2 diabetes [52]. Another M1 hub protein, SORBS1 (Fig. 6), which functions in insulin signaling, has also been associated with obesity and type 2 diabetes [51]. The finding of obesity and diabetes-associated FTO and SORBS1 as top hub proteins in AD-related M1 module is consistent with increasing evidence indicating the presence of shared pathways in the pathogenesis of AD, obesity, and diabetes [65].

M2, a 152-member module with positive correlation to AD phenotypes (Fig. 4), was highly enriched with GO categories, enzymes, and hub proteins linked to metabolic processes and pathways (Fig. 6 and Additional file 6: Table S6). The most prominent feature of this module is the presence of over 40 proteins that function in the carboxylic acid metabolism with serine racemase (SRR) and enolase 1 (ENO1) as top hub proteins (Fig. 6 and Additional file 5: Table S5). SRR, an enzyme for catalyzing the conversion of L-serine to D-serine (an essential co-agonist of the NMDA receptor) [15], was up-regulated by more than two folds in AD (Additional file 2: Table S2), which may lead to over-activated NMDA receptors, thereby contributing to AD pathophysiology. The M2 module was also highly enriched with proteins involved in the unsaturated fatty acid metabolic process (ACAA1, ACOX1, EPHX2, HSD17B4, LTA4H, PTGDS, PTGR1, PTGR2, GSTM2, GSTM3, GSTP1, and MIF), highlighting a link between dysregulated unsaturated fatty acid metabolism and AD pathophysiology. Furthermore, the M2 module was also significantly enriched with regulators of lipid metabolism (AGK, ACAA2, ALDH3A2, ANXA1, ANXA2, ANXA4, ANXA5, ASAH1, APPL2, DBI, ESYT1, GM2A, HADHA, INPP1, PAFAH1B3, ERLIN2, SLC44A2, PCYT2, PLCD3, and PRDX6) with annexin A5 (ANXA5) as a top hub protein (Fig. 6 and Additional file 5: Table S5). These findings provide new insights into the molecular basis of dysregulated lipid homeostasis in AD brain [26, 60].

The identified top M2 hub proteins also include all three members of the ezrin-radixin-moesin (ERM) family, ezrin (EZR), radixin (RDX), and moesin (MSN), which were up-regulated in AD (Fig. 6 and Additional file 2: Table S2), suggesting a role of ERM proteins in AD. The ERM proteins are FERM (4.1 protein, ezrin, radixin, moesin) domain-containing proteins that function as plasma membrane–cytoskeleton linkers to regulate membrane dynamics, cell adhesion, migration, signal transduction, and immune response [64]. Interestingly, another FERM domain-containing protein, FERMT2, was also identified as an up-regulated M2 hub protein with high intramodular connectivity (Fig. 6, Additional file 2: Table S2, and Additional file 5: Table S5). Our finding, together with the reports of FERMT2 as a genetic risk factor for AD [22] and a modulator of APP metabolism and tau neurotoxicity [16, 72], supports the involvement of FERMT2 in AD pathogenesis.

M15, a 57-member module positively correlated with AD phenotypes (Fig. 4), was significantly enriched with GO terms and proteins linked to immune response (ALCAM, ALAD, GAPDH, CYB5R3, DDX3X, CAPN1, PPIA, PYGB, EIF2AK2, CAB39, TTR, PDAP1, HIST1H2BK, QARS, VAPA, and PNP) with GAPDH, PPIA, CYB5R3, and PYGB as top hub proteins (Fig. 6 and Additional file 6: Table S6). Our finding of PPIA, CYB5R3, and PYGB, which are associated with neutrophil activation in immune response [31], as up-regulated M15 hub proteins (Fig. 6 and Additional file 2: Table S2) supports a role of neutrophil-dependent immune response in AD pathophysiology [91]. The enrichment of several aminoacyl-tRNA synthetases for protein translation (SARS, QARS, and NARS) in this module (Fig. 6) is in agreement with AD-associated, protein translation alteration identified in the M1 module.

M16, a 55-member, AD-positively correlated module (Fig. 4), was significantly enriched with GO categories and proteins linked to inflammatory response (C3, FHL1, A2M, CD44, FN1, HP, SERPINA1, and SERPINA3) with complement C3 and alpha-2-macroglobulin (A2M) as top hub proteins (Fig. 6 and Additional file 6: Table S6). Our finding of C3 and A2M, two key components of inflammatory response [42, 63], as up-regulated hub proteins in AD brain (Fig. 6 and Additional file 2: Table S2) supports their potential as candidate AD biomarkers [39] and the link between neuroinflammation and AD pathogenesis [14]. Another key molecular feature of M16 module is significant overrepresentation of extracellular matrix proteins (COL6A1, COL6A2, COL6A3, COL18A1, LAMA5, FLNA, and FLNB) as hub proteins (Fig. 6), providing evidence for the involvement of extracellular matrix dysfunction in AD [49].

M19, a 48-member module with positive correlation to AD phenotypes (Fig. 4), was highly enriched with GO terms and proteins linked to small GTPase-mediated trafficking and signaling (RAB1A, RAB1B, RAB3C, RAB4A, RAB4B, RAB6B, RAB8B, RAB10, RAB12, RAB14, RAB23, RAB35, ARF4, and ARF5) with Rab GTPases RAB1A, RAB1B, RAB4A, and RAB4B as top hub proteins (Fig. 6 and Additional file 6: Table S6). The enriched Rab and ARF GTPases function as key regulators of the following trafficking pathways: ER-to-Golgi transport (RAB1A and RAB1B), synaptic vesicle exocytosis and neurotransmitter release (RAB3C), endosome-to-plasma membrane recycling (RAB4A, RAB4B, RAB23, and RAB35), intra-Golgi traffic and endosome-to-Golgi transport (RAB6B), *trans*-Golgi network (TGN)-to-plasma membrane transport (RAB8B, RAB10, RAB12, RAB14, ARF4, and ARF5), Golgi-to-ER retrograde transport (ARF4 and ARF5), and autophagosome formation (RAB1A, RAB1B, RAB12, and RAB23) [24, 34, 75]. In addition, this module also contained endocytic trafficking regulators, NECAP1 and SORT1 (Fig. 6). The enrichment of the vesicular trafficking machinery components in the AD-correlated M19 module highlights the dysregulation of multiple trafficking pathways in AD brain.

M4, a 119-member module with negative correlation to AD phenotypes (Fig. 4), was highly enriched with GO categories and proteins linked to mitochondrial processes (Fig. 7, Additional file 5: Table S5, and Additional file 6: Table S6), including the mitochondrial contact site and cristae organizing system (MICOS) components (IMMT/MIC60 and CHCHD3/MIC19), mitochondrial import machinery components (TOMM70 and TIMM9), mitochondrial carrier system components (SLC25A3 and SLC25A12), mitochondrial inner membrane fusion GTPase OPA1, electron transport chain subunits (MT-ND5, NDUFA12, NDUFS5, NDUFS7, SDHA, COX4I1, COX5B, COX6C), and mitochondrial ATP synthase subunits **(**ATP5A1, ATP5B, and ATP5J**)**. The identification of IMMT and SLC25A3 as down-regulated hub proteins in AD brain (Fig. 7 and Additional file 2: Table S2) reveals a previously unrecognized role of the MICOS system and mitochondrial carrier system in AD pathophysiology. The M4 module was also enriched with GO categories and proteins associated with synaptic structure and function (Fig. 7, Additional file 5: Table S5, and Additional file 6: Table S6), including key presynaptic proteins involved in synaptic vesicle trafficking and neurotransmitter release (SNAP25, STX1B, SYP, SV2B, VAMP1, RPH3A, and RAB3GAP2), glutamate receptor subunits (GRIA2/GluR2 and GRIA4/GluR4), GABA_A_ receptor β1 subunit (GABRB1), and postsynaptic density proteins (SYNGAP1, DLG1/SAP97, DLG3/SAP102, and SRGAP3). The finding of these synaptic proteins in the AD-down-regulated M4 module is consistent with a loss of synaptic function in AD [28].

M5, a 106-member module negatively correlated with AD phenotypes (Fig. 4), was highly enriched with GO terms and proteins linked to oxidative phosphorylation (MT-ND3, NDUFA2, NDUFA7, NDUFAB1, NDUFB11, NDUFS1, NDUFS8, CYC1, UQCRC1, COX5A, COX6B1, and ATP5D) with MT-ND3 and UQCRC1 as top hub proteins, synaptic cell adhesion (CADM2/SynCAM2, CADM3/SynCAM3, NCAM1, LSAMP, NTM, OPCML) with CADM2/SynCAM2 and LSAMP as top hub proteins, synaptic vesicle exocytosis (STX1A, CPLX2, and SYT12), and signal transduction (PPP1CB, MARK2, PAK1, PRKCE, PRKCG, SLK, STK39, RHOA, RHOB, RHOC, RHOG, and CDC42) with PPP1CB, RHOA, and STK39 as top hub proteins (Fig. 7, Additional file 5: Table S5, and Additional file 6: Table S6). These findings further support the involvement of impaired mitochondrial and synaptic functions and dysregulated signaling in AD pathophysiology.

M10, a 62-member module with the most significant negative correlation to AD phenotypes (Fig. 4), was highly enriched with ion-transporting ATPases, such as Na^+^/K^+^ ATPase subunits (ATP1A2, ATP1B1, and ATP1B2) for establishing the electrochemical gradients of Na and K ions across the plasma membrane and the H^+^-transporting, vacuolar ATPase subunit ATP6V1G2 for lysosomal acidification (Fig. 7 and Additional file 6: Table S6), supporting a loss of brain cell ion homeostasis in AD pathogenesis [20, 81]. The M10 module was also significantly enriched with GO terms and proteins linked to transmembrane transport and vesicle-mediated transport (Fig. 7 and Additional file 6: Table S6), including mitochondrial protein import (MTX2), endocytosis (DNM2, DNM3), endosome-to-lysosome trafficking and synaptic vesicle biogenesis (AP3D1), ER-to-Golgi transport (BCAP31), intra-Golgi trafficking (USO1, NAPA, NAPG), TGN-to-plasma membrane transport (AP1M1), endosome-to-plasma membrane recycling (SNX27), and autophagy (GABARAPL2, RTN3, and UBQLN1). The enrichment of these transport machinery components in the AD-down-regulated M10 module indicates impairment of multiple transport pathways in AD brain.

M11, a 62-member module negatively correlated with AD phenotypes (Fig. 4), has heterotrimeric G-protein subunits (GNAI1, GNAI2, GNAI3, and GNAO1) and Src family of tyrosine kinases (FYN and YES1) as top hub proteins (Fig. 7), highlighting the involvement of altered intracellular signaling in AD pathophysiology. In addition, the M11 module was significantly enriched with GO terms and proteins linked to regulation of actin cytoskeleton (CRMP1, CRMP4/DPYSL3, ABI1, FSCN1, and WASF1) and microtubule cytoskeleton (TUBAL3, TBCB, NDRG1, NDRG2, SHTN1, and SNCG) (Fig. 7 and Additional file 6: Table S6), consistent with impaired actin and microtubule dynamics in AD brain [5, 25]. The association of the retromer complex components (SNX1 and SNX2) with the M11 module supports a link between retromer dysfunction and AD pathogenesis [73].

M12, a 61-member module with negative correlation to neurofibrillary tangle pathology but not amyloid plaque pathology (Fig. 4), is characterized by highly significant enrichment of GO terms and proteins linked to myelin sheath (CNP, MAG, MBP, OMG, PLP1, MOG, PMP2, CLDN11, and ERMN) and the organization of paranodal and juxtaparanodal regions of axon at the node of Ranvier (MAG, ERMN, CNTNAP1, CNTN2, and KCNA2) with CNP, MAG, OMG, and PLP1 as top hub proteins (Fig. 7 and Additional file 6: Table S6). These results, together with our finding of OMG and PLP1 as down-regulated hub proteins in AD (Fig. 7 and Additional file 2: Table S2), support the involvement of myelin degeneration, impaired myelin-axon interactions, and node of Ranvier dysfunction in AD pathogenesis [8]. The M12 module was also significantly enriched with neurofilament proteins (NEFL, NEFM, and INA) and microtubule-binding proteins involved in the control of microtubule polymerization or stabilization (CRYAB, MAPRE1, DST, CRMP2/DPYSL2, CLASP2, and MAP1B) and axonal transport (DCTN1 and DCTN4), indicating an association of impaired neurofilament and microtubule functions with Tau aggregation in AD. Our finding of BIN1, the second most prevalent genetic risk factor for sporadic AD [22], as a member of the M12 module with negative correlation to neurofibrillary tangle pathology (Fig. 7) is consistent with recent evidence indicating that BIN1 negatively regulates the propagation of Tau pathology [13].

M13 is a 61-member module down-regulated in AD (Fig. 5) with negative correlation to AD phenotypes (Fig. 4). More than one-third of proteins in this module are associated with hydrolase activity, represented by top hub proteins such as deubiquitinating enzyme OTUB1, small GTPase RAC1, adenosylhomocysteinase-like proteins AHCYL1 and AHCYL2, protein tyrosine phosphatase PTPRZ1, and platelet-activating factor acetylhydrolase subunit PAFAH1B1 (Fig. 7 and Additional file 5: Table S5). In addition, the M13 module was also significantly enriched with GO terms and proteins linked to organization of the actin cytoskeleton (ARPC1A, CFL1, CFL2, CTTN, RAC1, PAFAH1B1, NF1, SPTAN1, and SPTBN2) and microtubule cytoskeleton (MAPRE3, KLC2, NIT2, TBCA, TUBA8, MAP7D1) (Fig. 7 and Additional file 6: Table S6), supporting the involvement of impaired actin and microtubule dynamics in AD pathophysiology [5, 25].

## Discussion

This study shows that integration of quantitative proteomics, differential expression analysis, and co-expression network analysis provides a useful approach for gaining systems-level insights into AD pathogenesis. A critical step in quantitative proteomic analysis is sample preparation, which is a key determinant of the quality of generated proteomic data set. Previous proteomic studies of AD brains used detergent-free, protein extraction with a chaotropic reagent such as urea, which is unable to completely solubilize brain tissue and extract all proteins [3, 56, 83, 87]. To overcome this limitation, we used the strong detergent SDS for complete solubilization and extraction of proteins followed by the filter-aided sample preparation procedure [85, 87] to obtain high-purity peptides for LC-MS/MS-based quantitative proteomic analysis. Our proteomic results support that the SDS-based, filter-aided sample preparation method is highly effective for achieving high proteome coverage and reliable measures of protein expression levels in human AD and control brain tissues.

Differential expression analysis, which compares expression levels for individual proteins between AD and control groups, is a commonly used method in proteomic studies to identify AD-associated protein changes [3, 56, 59]. Using this method, we have identified 487 differentially expressed proteins with significantly altered protein levels (> 1.3-fold change; *P* < 0.05) in AD versus control, including 262 up-regulated proteins and 225 down-regulated proteins involved in multiple biological processes. The identification of a wide spectrum of protein alterations is consistent with the multifactorial and complex etiology of AD. Our identified differentially expressed proteins include 322 novel proteins that are not previously known to be altered in AD (Additional file 3: Table S3), providing new insights into protein changes in AD brain. Due to the small sample size, this study is expected to have false positives as well as well as false negatives. The identified differentially expressed proteins in AD have a false discovery rate of < 11% based on the estimation by *q* values (Additional file 2: Table S2). Therefore, our findings will need to be confirmed in larger samples. The independent validation of altered expression in AD of two identified novel proteins, STK39 and DIABLO/Smac, by Western blot analysis highlights the robustness of our label-free quantitative proteomic analysis.

In contrast to differential expression analysis which determines expression changes of single proteins independently, co-expression network analysis relates proteins to each other using pairwise correlation relationships between protein expression profiles to illuminate higher-order molecular organization and define modules of co-expressed proteins that are functionally related and/or coordinately regulated [45, 90, 92]. Using this network analysis, we have identified 11 disease-associated, protein co-expression modules that are significantly correlated with AD phenotypes, including 5 positively correlated modules (M1, M2, M15, M16, and M19) and 6 negatively correlated modules (M4, M5, M10, M11, M12, and M13). The identified, AD-associated modules reveal a number of previously unrecognized co-expression relationships among proteins involved in distinct biological processes and provide a novel view of cellular mechanisms. For example, the M1 module shows that proteins controlling various processes of proteostasis (e.g., protein translation, protein folding, and proteasome-mediated degradation) and RNA homeostasis (e.g., RNA processing, transcription initiation, mRNA modification and stability) are highly connected at a co-expression level (Fig. 6, Additional file 5: Table S5, and Additional file 6: Table S6), indicating coordinate control or interactions among these different processes. The M12 module reveals a strong co-expression relationship linking myelin proteins, neurofilament proteins, and axonal proteins involved in microtubule-based transport (Fig. 7 and Additional file 6: Table S6), highlighting the glia-neuron interactions and coupling between myelin and axonal processes. The M19 module uncovers a previously unknown co-expression relationship connecting Rab GTPases, ARF proteins, and other key regulators of various intracellular membrane trafficking processes (Fig. 6 and Additional file 6: Table S6), suggesting co-regulation of multiple trafficking processes and their involvement in AD pathophysiology. Further studies of the identified protein co-expression relationships and their regulation will advance our knowledge of the cellular mechanisms governing coordinate control and concerted actions of various biological processes in health and Alzheimer’s disease.

Our proteomics-driven network analysis has generated a molecular blueprint of dysregulated protein networks in AD brain and has uncovered many new proteins and pathways in processes implicated in AD, including altered proteostasis, RNA homeostasis, immune response, neuroinflammation, synaptic transmission, vesicular transport, cell signaling, cellular metabolism, lipid homeostasis, mitochondrial dynamics and function, cytoskeleton organization, and myelin-axon interactions. The identified hub proteins of AD-associated protein network modules are particularly useful for biomarker and therapeutic development, as hub proteins are often key drivers of disease-related co-expression modules or key determinants of module function [12, 27, 33, 46, 54, 82, 88]. Our finding that the identified top hub proteins can serve as a molecular signature for differentiating AD and control cases (Fig. 8c) supports their potential as novel AD biomarkers. Furthermore, the hub proteins of AD-related modules uncovered in this study provide attractive drug targets for developing novel therapeutics to shift disease-specific changes of protein networks and cellular functions back to their normal range.

## Conclusions

In summary, our integrated proteomics and network analysis provides a systems-level view of proteome changes in AD brain and uncovers disease-associated protein network alterations in AD. The identified AD-related network modules and their hub proteins generate new insights into the pathogenesis of sporadic AD. Our findings suggest new targets and biomarker candidates for AD diagnostic development and therapeutic intervention.

## Additional files

Additional file 1: **Table S1.** Demographic and neuropathological data of human AD patient and control cases. For each case, the age, gender, disease status, age at onset, disease duration, Braak stage, CERAD neuritic plaque score, frontal cortex neuritic plaque frequency, ApoE genotype, and postmortem interval (PMI) are provided. (XLSX 10 kb)

Additional file 2: **Table S2.** Differential expression analysis of protein abundances in AD and control brains. List of all proteins with complete abundance data in AD and control brains is provided with their fold changes, *P* values, and *q* values. Differentially expressed proteins with significantly altered protein abundances (> 1.3-fold change; *P* < 0.05) in AD versus control are indicated in bold and also provided in separate tabs. (XLSX 660 kb)

Additional file 3: **Table S3.** List of novel proteins with altered abundances in AD identified in the present study. The fold changes of protein abundances in AD versus control are provided with corresponding *P* values and *q* values. (XLSX 51 kb)

Additional file 4: **Table S4.** Gene ontology (GO) term enrichment for differentially expressed proteins in AD. The enriched GO terms with associated *P* values (Benjamini-Hochberg FDR corrected) for biological processes, cellular compartments, and molecular functions are provided in separate tabs. (XLSX 35 kb)

Additional file 5: **Table S5.** Protein co-expression network analysis by WGCNA. Network analysis of the entire proteomic data set from all AD and control cases identified 24 network modules, M1 to M24, coded by different colors according to the convention of WGCNA. Proteins that were not assigned to any module were coded by the color grey in M0. The complete list of proteins in each module and their module membership values (kME) are provided. (XLSX 724 kb)

Additional file 6: **Table S6.** Gene ontology (GO) term enrichment for proteins in WGCNA modules. The enriched GO terms with associated *P* values (Benjamini-Hochberg FDR corrected) for biological processes, cellular components, and molecular functions are provided in separate tabs. (XLSX 147 kb)
